# Tauroursodeoxycholic Acid (TUDCA)—Lipid Interactions and Antioxidant Properties of TUDCA Studied in Model of Photoreceptor Membranes

**DOI:** 10.3390/membranes11050327

**Published:** 2021-04-29

**Authors:** Michał J. Sabat, Anna M. Wiśniewska-Becker, Michał Markiewicz, Katarzyna M. Marzec, Jakub Dybas, Justyna Furso, Paweł Pabisz, Mariusz Duda, Anna M. Pawlak

**Affiliations:** 1 Department of Biophysics, Faculty of Biochemistry, Biophysics and Biotechnology, Jagiellonian University, 30-387 Krakow, Poland; michal.sabat@student.uj.edu.pl (M.J.S.); anna.m.wisniewska@uj.edu.pl (A.M.W.-B.); justyna.furso@uj.edu.pl (J.F.); pawel.pabisz@doctoral.uj.edu.pl (P.P.); mariusz.z.duda@gmail.com (M.D.); 2 Department of Computational Biophysics and Bioinformatics, Faculty of Biochemistry, Biophysics and Biotechnology, Jagiellonian University, 30-387 Krakow, Poland; m.markiewicz@uj.edu.pl; 3 Jagiellonian Centre for Experimental Therapeutics, Jagiellonian University, 30-348 Krakow, Poland; katarzyna.marzec@uj.edu.pl (K.M.M.); jakub.dybas@uj.edu.pl (J.D.)

**Keywords:** bile acids, liposome, photoreceptor membrane, lipid peroxidation, antioxidant, electron paramagnetic resonance

## Abstract

Tauroursodeoxycholic acid (TUDCA), a hydrophilic bile acid containing taurine conjugated with the ursodeoxycholic acid (UDCA), has been known and used from ancient times as a therapeutic compound in traditional Chinese medicine. TUDCA has recently been gaining significant interest as a neuroprotective agent, also exploited in the visual disorders. Among several mechanisms of TUDCA’s protective action, its antioxidant activity and stabilizing effect on mitochondrial and plasma membranes are considered. In this work we investigated antioxidant activity of TUDCA and its impact on structural properties of model membranes of different composition using electron paramagnetic resonance spectroscopy and the spin labeling technique. Localization of TUDCA molecules in a pure POPC bilayer has been studied using a molecular dynamics simulation (MD). The obtained results indicate that TUDCA is not an efficient singlet oxygen (^1^O_2_ (^1^Δ_g_)) quencher, and the determined rate constant of its interaction with ^1^O_2_ (^1^Δ_g_) is only 1.9 × 10^5^ M^−1^s^−1^. However, in lipid oxidation process induced by a Fenton reaction, TUDCA reveals substantial antioxidant activity significantly decreasing the rate of oxygen consumption in the system studied. In addition, TUDCA induces slight, but noticeable changes in the polarity and fluidity of the investigated model membranes. The results of performed MD simulation correspond very well with the experimental results.

## 1. Introduction

**Figure 1:**
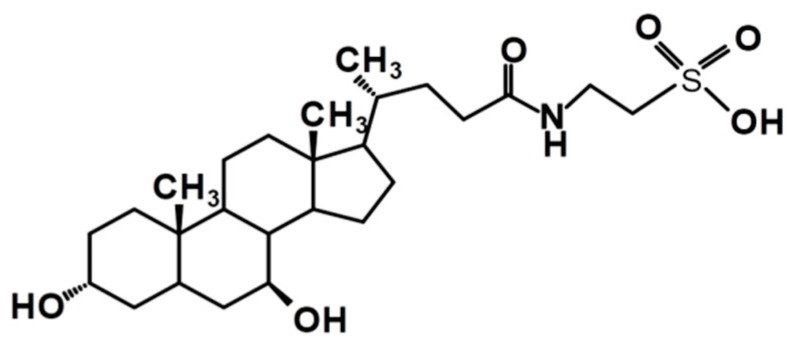
Chemical structure of tauroursodeoxycholic acid, TUDCA.

Tauroursodeoxycholic acid (TUDCA) composed of taurine conjugated with the ursodeoxycholic acid (UDCA), together with its precursor UDCA, belong to the most hydrophilic bile acids [1,2] (Figure 1).

Although present in the biliary bile acids of a number of vertebrates in a small amount (below 1–5%), these bile acids constitute major components of a bear bile, in which their level reach up to 75% [3,4]. In recent years TUDCA and, although in a lesser extent, UDCA, have been gaining growing attention due to their cytoprotective, neuroprotective and antiapoptotic properties [5,6,7,8]. However, TUDCA has been known and used from ancient times as a therapeutic compound in traditional Chinese medicine (TCM) [9,10]. According to TCM dried bear bile helps in detoxification, inflammation, pain reduction and to dissolve kidney stones and gallstones or to improve vision [10]. In recent decades the UDCA and especially the TUDCA were successfully applied as an efficient potent antiapoptotic and cytoprotective agents in numerous models of various neurodegenerative diseases such as amyotrophic lateral sclerosis, Alzheimer, Parkinson or Huntington disease [6,11,12,13]. In addition, TUDCA was shown to be particularly effective in protection of photoreceptor cells against photodamage regardless of the mechanism behind [9,14,15,16,17]. In both retinal damage models: murine model of retinitis pigmentosa (rd10 strain) and excessive light induced retinal damage (LIRD) TUDCA supplementation greatly slowed retinal degeneration [9,14]. The therapeutic effect resulted in preservation of function and morphology of photoreceptor cells in retina [9,14,15,16,17]. Among the proposed mechanism of cytoprotective action of TUDCA we may include the inhibition of classic apoptosis pathways [18], decrease of endoplasmic reticulum stress and protein aggregation [19,20], stabilization of mitochondrial membranes [21,22], prevention of DNA damage [23], and stimulation of antioxidant mechanisms through activation of the nrf2 transcription factor [24,25]. The most recent data demonstrated that if ARPE–19 cells were exposed to H_2_O_2_ in the presence of TUDCA, the bile acid increased cells viability and enhanced their antioxidant capacity by increasing the level of glutathione and upregulating the expression of antioxidant genes [25]. Earlier studies showed antioxidant activity and membrane stabilizing effect of TUDCA also in non-cellular models, however these studies focused on the interaction of UDCA and TUDCA with other components of the bile [26,27,28]. In light of these reports, interaction of TUDCA with lipid membrane and/or their components seems to be one of the key mechanisms in its neuroprotective action.

Light absorption and first stages of the visual signal transduction take place in photoreceptor outer segments (POSs) [29]. In case of rod type cell POS are built in a form of stack of membranous disks enveloped with a plasma membrane (PM) [30]. Lipid composition of PM and membranes of newly synthesized disks laying at the base of the POS and disks in its apical tip visibly change—a major difference exists in the abundance of cholesterol (Ch) and polyunsaturated fatty acids [31,32]. These differences strongly affect the physical properties of the membrane and are associated with the process of light detection, activation and deactivation of rhodopsin (Rh) [32]. Significant differences in cholesterol content may affect interaction of TUDCA with the membranes and thus also modify its protective functions [28]. Even though the way TUDCA interplays with the membranes and their components has been investigated, the reported results are very often contradictory. Thus, the investigation of the effect of TUDCA on model membranes with low and high Ch/phospholipid ratio and possible ability to stabilize membranes is of particular importance. In our study we analyzed whether the protective effect of TUDCA against photoreceptor photodamage could be facilitated to some extent by its structural effects on the membrane stemming from its chemical and physical properties.

## 2. Materials and Methods

### 2.1. Lipids and Spin Labels

1-palmitoyl–2–oleoyl–sn–glycerol–3–phosphocholine (POPC), 1,2–dioleoyl–sn–glycerol–3–phosphocholine (DOPC), 1,2–dimyristoyl–sn–glycerol–3–phosphocholine (DMPC), 1,2–dimyristoyl–sn–glycerol–3–phosphoethanolamine (DMPE), 1–palmitoyl–2–docosahexaenoyl–sn–glycerol–3–phosphocholine ((16:0)(22:6) PC)), 1–palmitoyl–2–oleoyl–sn–glycerol–3–phosphoethanolamine (POPE), egg-yolk sphingomyeline (SM), cholesterol and spin labels 1–palmitoyl–2–stearoyl–(5–doxyl)–sn–glycerol–3–phosphocholine, 1–palmitoyl–2–stearoyl–(7–doxyl)–sn–glycerol–3–phosphocholine, 1–palmitoyl–2–stearoyl–(10–doxyl)–sn–glycerol–3–phosphocholine, and 1–palmitoyl–2–stearoyl–(16–doxyl)–sn–glycerol–3–phosphocholine were purchased from Avanti Polar Lipids (Alabaster, USA). mHCTPO was a gift from Prof. H. J. Halpern (University of Chicago, Chicago, IL, USA).

### 2.2. Preparation of Liposomes

Two models of liposomes were prepared. For structural measurements involvbruing electron paramagnetic resonance (EPR) technique simpler models were used consisting of POPC and DMPE (6:1 molar ratio) with two different concentration of cholesterol (5 or 40 mol%). For oxygen consumption measurements more complex liposome models were employed mimicking the membranes of POS. In this case, POPE instead of DMPE was used. Apart from POPC and POPE, a peroxidizable phospholipid containing docosahexaenoic acid ((16:0)(22:6) PC) was added in the concentration of 35 mol%. At the end, the molar ratio between POPC and POPE was 1:1. Additionally, cholesterol was present in the concentration of 5 mol %. The lipid composition was therefore similar to that of the old disks in POS [32]. The liposomes were prepared by the following method. Briefly, chloroform solutions of lipids (containing 10 μmol of total lipids), ethanol solution of TUDCA or UDCA (5 mol %, if applicable), and doxyl spin labels (1 mol %, if applicable) were mixed, organic solvent was evaporated with a stream of nitrogen, and the lipid film on the bottom of the test tube was thoroughly dried under reduced pressure (about 0.1 mm Hg) for 12 h. A phosphate buffered saline (usually 0.5 mL) was added to the dried film at the temperature well above the lipid phase transition temperature (T_M_) and vortexed vigorously. Then, the multilamellar liposome suspension for structural measurements underwent five freeze–thaw cycles, after which it was centrifuged at 14,000× *g* for 15 min at 4 °C, and the resulting pellet was used for EPR measurements. In case of oxygen consumption measurements, multilamellar liposome suspension was not centrifuged and the final lipid concentration was 10 mM.

### 2.3. Singlet Oxygen Quenching Measurements

To determine rate constants of the interactions of singlet oxygen with the bile acids studied, time-resolved singlet oxygen phosphorescence at 1270 nm was measured as a function of the quencher concentration. Tetraphenylporphyrine (TPP) was used as an efficient sensitizer with quantum yield of singlet oxygen (^1^O_2_, ^1^Δ_g_) generation up to 0.63 in benzene [33,34], and its absorbance was adjusted to 0.06 at the excitation wavelength (λ = 590 nm). Sensitizer in a mixture of chloroform and DMSO–d6 (1:1, v/v) placed in a quartz fluorescence cuvette (QA–1000; Hellma, Mullheim, Germany) was excited with 590 nm light generated by an integrated nanosecond DSS Nd:YAG laser system (NT242–1k–SH/SFG; Ekspla, Vilnius, Lithuania). The near-infrared singlet oxygen phosphorescence was measured perpendicularly to the excitation beam in a photon-counting mode using a thermoelectric cooled NIR PMT module (H10330-45; Hamamatsu Photonics, Hamamatsu C Japan). Measurements were repeated with increasing concentrations of UDCA or TUDCA. Data analysis, including first-order ^1^O_2_ (^1^Δ_g_) phosphorescence decay fitted by the Levenberg–Marquardt algorithm, was performed by custom-written software.

### 2.4. EPR Oximetry

To determine Fenton reaction-induced oxygen uptake rate in peroxidizable liposomes in the presence of studied bile acid (5 mol%), electron paramagnetic resonance oximetry with the mHCTPO (0.1 mM) spin probe was utilized. Samples (final volume of 200 µL) for oxygen uptake rate measurements consisted of previously prepared liposomes (150 µL), spin probe (20 µL, final concentration 0.1 mM), ferrous citrate (10 µL, final concentration 25 μM), and ascorbic acid (20 μL, final c oncentration 200 μM) solutions. Measurements were carried out in a flat quartz cell placed in the EPR resonant cavity as previously described [35,36]. EPR measurements were performed using the following instrument parameters: microwave power 1.06 mW, modulation amplitude 0.006 mT, scan width 0.3 mT, and scan time 5.2 s using a Bruker EMX-AA 1579 EPR spectrometer (Bruker BioSpin, Rheinstetten, Germany).

### 2.5. EPR Measurement of Structural Properties of Lipid Bilayers

T–PC, 5–PC, 7–PC, 10–PC, and 16–PC are phospholipid spin labels that have a nitroxide free radical moiety responsible for the EPR signal attached to the polar headgroup, or to the 5th, 7th, 10th, or 16th carbon atom in the acyl chain, respectively. Therefore, information is obtained from different regions of the membrane: the water-membrane interphase (T-PC), the region close to the polar headgroups (5- and 7-PC) and the membrane center (10- and 16-PC). The EPR measurements were performed with Bruker EMX spectrometer (Bruker, BioSpin, Rheinstetten, Germany) equipped with a temperature control unit (EMX ER 4141 VT). The suspension of spin labeled liposomes was placed in a gas permeable capillary (i.d. 0.7 mm) made of TPX and located inside the EPR dewar insert in the resonant cavity of the spectrometer. The sample was deoxygenated with nitrogen gas (about 10 min), which was also used for temperature control. For measurements of lipid order (S parameter), the EPR spectra were recorded at 310 K whereas for polarity measurements (2A_ZZ_), samples were frozen to 120 K using liquid nitrogen vapor.

### 2.6. Molecular Dynamics Simulation

The model system used in this study consisted of 240 POPC, 16 TUDCA and 17,800 water molecules. Additionally, 16 Na^+^ ions were added to compensate the charges of anionic sulfonate groups of TUDCA. The initial models of TUDCA molecules were constructed using the Pymol program [37]. In order to check preferential location of TUDCA molecules in the phospholipid bilayer we assumed the same probability of their location in the lipid and in the water phases. Eight TUDCA molecules were placed in the water phase near the interphase, parallel to the membrane surface. The other eight were placed in the bilayer core, parallel to the POPC molecules; four TUDCA molecules with the sulphate group directed to the water phase and four TUDCA molecules with the sulphate group directed to the hydrophobic bilayer core. The bilayer system was constructed using the CHARMM-GUI server [38]. For POPC and ions CHARMM36 parameters [39], for TUDCA CHARMM/CGenFF [40] and for water, TIP3P parameters [41] were used. The LINCS algorithm was applied to all CH, OH and NH bonds of the POPC, TUDCA and water molecules and the time step was set at 2 fs. The electrostatic interactions were evaluated using the particle-mesh Ewald (PME) summation method [42]. The three-dimensional periodic boundary conditions with the usual minimum image convention and a cut-off of 12 Å were used. Simulations were carried out at a constant temperature of 310 K = 37 °C, which is above the main phase transition temperature for a pure POPC bilayer (−5 °C) [43], and a constant pressure (1 atm). The temperature was controlled using Nosé–Hoover thermostat [44,45], separately for the solute and solvent, and the pressure was controlled by the Parrinello–Rahman method [46]. The applied pressure was controlled semi–isotropically. The model system was MD-simulated for 500 ns. Average values of quantities obtained in MD simulations are time averages calculated from the final 200 ns fragment of the trajectory. For average values of hydrogen bonds, the errors are standard deviation estimates.

The orientational disorder of acyl chains in the membrane was calculated using molecular order parameter S_mol_. S_mol_ for the nth segment of an acyl chain is defined through:

S_mol_ = 0.5 × (3cos (θ_n_) − 1) (1)

where θ_n_ is the instantaneous angle between the nth segmental vector (i.e., the (C_n − 1_, C_n + 1_) vector linking n − 1 and n + 1 carbon (C) atoms in the acyl chain) and the bilayer normal with corrections for sp2 hybridized carbon atoms linked by the double bond [47,48]. The errors in average values of S_mol_ are standard errors and were calculated over 5 blocks, with each being a 40 ns fragment of the productive trajectory.

### 2.7. Isolation of Detergent–Resistant (DRM) and Detergent–Soluble (DSM) Membrane Fractions

For isolation of detergent-resistant lipid rafts (also called detergent-resistant membrane (DRM) fraction) we used a simplified procedure similar to that described previously [49]. Multilamellar liposomes consisting of equimolar mixtures of unsaturated lipid (DOPC 1.5 µmol) and saturated lipids (SM, 1 µmol, and DMPC, 0.5 µmol), and cholesterol (0.5 µmol) containing 0.2 µmol TUDCA were suspended in PBS (4.8 mg of lipids/mL). Then, the suspension was treated with 1% Triton X-100 for 30 min at 4 °C and centrifuged for 30 min at 4 °C with an Eppendorf bench centrifuge (at 16,000× *g*). Then lipids were extracted from DSM and DRM fractions using the Folch method [50]. DSM (500 μL) or resuspended DRM samples (500 μL) were mixed with 800 μL of chloroform/methanol mixture (2/1 v/v), well vortexed, and centrifuged for 3 min at 16,000× *g*. The lower chloroform portion was dried under nitrogen and then lipids left after drying were analyzed for the phospholipid, cholesterol, and TUDCA content by Raman spectroscopy.

### 2.8. Raman Spectroscopy (RS)

RS measurements of the TUDCA standard, DSM and DRM samples were conducted on a WITec confocal CRM alpha 300 Raman microscope with use of 488 nm excitation wavelength. The laser was coupled to the microscope via an optical fiber (50 μm diameter). A dry Olympus MPLAN objective (100×/0.90NA) was used. Spectral resolution was equal to 3 cm^−1^. The monochromator of the spectrometer was calibrated using radiation spectrum from a xenon lamp (WITec UV-light source, Ulm, Germany. Additionally, standard alignment procedure (single-point calibration) was performed before collection of spectra with use of Raman scattering line produced by a silicon plate (520.5 cm^−1^). Samples were transferred on a CaF_2_ slide and Raman spectra were collected from ten randomly chosen spots. Estimated acquisition time of a single spectrum was 5 s (10 accumulations, 0.5 s integration time). Laser power in the focus spot was equal to 10 mW. Data collection and analysis were performed using WITec (WITec Project Plus 5), Opus 7.2 and OriginPro 2018 programs.

## 3. Results and Discussions

### 3.1. Interaction of TUDCA with Singlet Oxygen

Quenching of singlet oxygen (^1^O_2_, ^1^Δ_g_) is one of the most apparent manifestations of antioxidant activity, understood as stopping or slowing down the oxidation process. Highly reactive singlet oxygen can initiate a chain lipid peroxidation reaction and oxidize other key cell components—proteins and nucleic acids [51]. Generation of singlet oxygen accompanies photosensitized reactions [52], which can take place in the outer retina, especially in the photoreceptor outer segments (POS) layer, was constantly exposed to intense light in the presence of endogenous sensitizers [53,54] and relatively high oxygen concentration [55]. Apoptotic photoreceptor damage and death in light-induced retinal degeneration (LIRD) mouse models may by induced by two different ways depending on light intensity and exposure time [56]. In one of them, transient accumulation of rhodopsin photobleaching products, including all-trans retinal (atRAL) [56], might be of great importance [54,57,58]. It has been shown that atRAL released from deactivated Rh might temporarily concentrate in POS membranes, as its reduction to all trans retinol is a limiting step in retinoid cycle [59]. AtRAL is photoreactive [60] and generates singlet oxygen with quantum yield of up to 30% upon excitation with 355 nm in benzene [61]. The amount of atRAL released even in the case of bleaching 0.5% of total amounts of Rh might reach toxic concentration in the retina [57,58].

**Figure 2:**
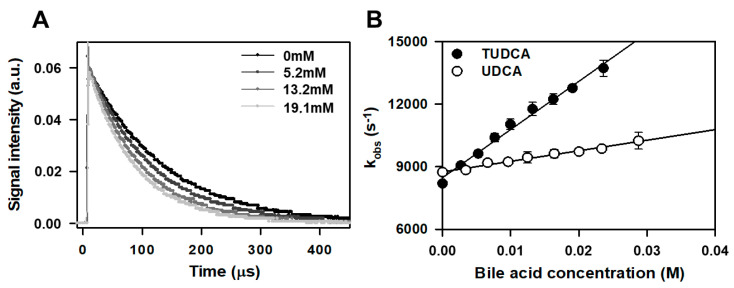
Singlet oxygen phosphorescence decays at 1270 nm acquired in TPP solution in DMSO–d6:CHCl_3_ (1:1, v/v) upon excitation with 590 nm laser pulse in the absence and in the presence of increasing concentration (5.2–19.1 mM) of TUDCA (**A**). Dependence of pseudo-first-order rate constant (s^−1^) of singlet-oxygen quenching on concentration of both bile acids studied TUDCA and UDCA (**B**).

To analyze the role of TUDCA in the protection of photoreceptors against photodamage, at first we investigated the basic antioxidant activity of TUDCA. Rate constant of interaction of TUDCA and its precursor UDCA with singlet oxygen has been determined in homogenous solution, while their impact on free radical-induced lipid peroxidation process has been studied in liposomes composed of peroxidizable lipids [62,63]. Detection of characteristic phosphorescence of singlet oxygen was performed in a homogenous solution of tetra-phenyl porphyrin (TPP) used as a ^1^O_2_ generator in a mixture of DMSO–d_6_:CHCl_3_ (1:1, v/v). Excitation of TPP with laser pulse at 590 nm generated singlet oxygen, which decayed with time constant (8.82 ± 0.43) × 10^3^ s^−1^. In the absence of bile acids, lifetime of singlet oxygen generated by TPP in the mixture of solvents used was 113.69 ± 5.34 µs. Representative results acquired in air-equilibrated solution of TPP excited with 590 nm in the absence and in the presence of increasing concentrations of TUDCA (5.2–19.1 mM) are shown in Figure 2A. Increase of rate constant of ^1^O_2_ phosphorescence decay at 1270 nm was observed in the presence of rising concentrations of both studied bile acids (Figure 2B).

The determined rate constant of singlet oxygen (^1^O_2_, ^1^Δ_g_) quenching by TUDCA and UDCA were (1.99 ± 0.32) × 10^5^ M^−1^s^−1^ and (0.39 ± 0.13) × 10^5^ M^−1^s^−1^, respectively (Table 1). Table 1 also includes the rate constants of interaction between singlet oxygen and several selected compounds of biochemical importance.

The difference between singlet oxygen quenching rates of both bile acids cannot be explained by the presence of taurine residue in the TUDCA molecule. Taurine itself interacts with singlet oxygen with very low efficiency, and the rate constant of singlet oxygen quenching by taurine does not exceed 1 × 10^3^ M^−1^s^−1^ [64] (Table 1). However, the presence of peptide bond in the TUDCA molecule may affect its quenching rate of singlet oxygen and may be the source of a significant difference between quenching rates determined for UDCA and TUDCA. The rate constant of peptide bond interaction with singlet oxygen was estimated to 1 × 10^5^ M^−1^s^−1^ [65]. Although not very high and rather negligible in the case of interaction of oligopeptides or proteins with ^1^O_2_ (^1^Δ_g_), it makes a significant contribution to the total singlet oxygen quenching rate of TUDCA. Even though the rate constant of singlet oxygen quenching by TUDCA is rather low, it should be noted that it is one of the highest interaction rates with singlet oxygen among sterols and comparable to that of phospholipids [66] (Table 1). However, it is still much lower than singlet oxygen quenching rate observed in case of biologically active endogenous antioxidants, e.g., α–tocopherol (Table 1). Therefore, singlet oxygen quenching cannot be considered as an important mechanism of amphiphilic bile acids antioxidant action. Due to the chemical structure of TUDCA, especially the lack of the C5=C6 double bond in the steroid ring structure, the interaction of TUDCA with singlet oxygen has mainly a physical character.

### 3.2. Reactivity of TUDCA towards Free Radicals

**Figure 3:**
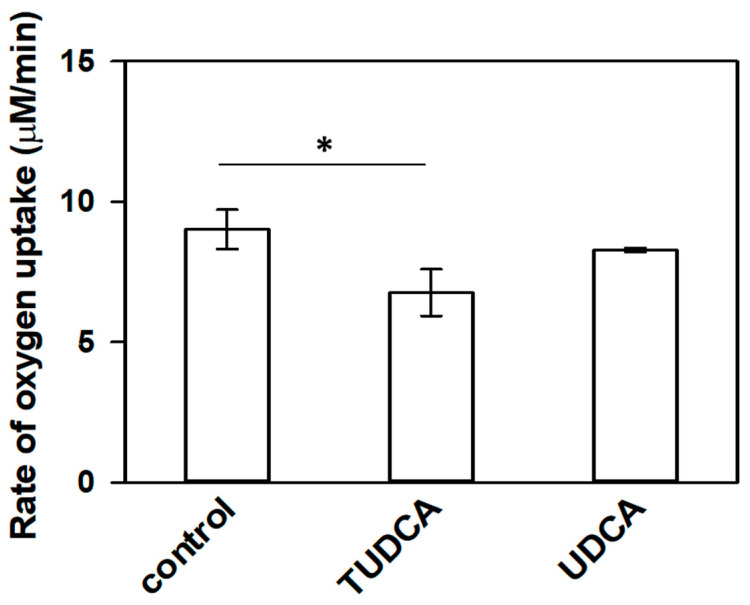
Oxygen consumption in liposomal samples composed of a mixture of synthetic lipids, naturally present in the photoreceptor membranes, exposed to free radical flux generated in Fenton reaction. Bars represent initial rates of oxygen uptake (μM/min). Decrease in oxygen consumption rate observed in the presence of TUDCA in liposomes statistically significant in comparison to control sample—liposomes not containing bile acid (* *p* < 0.05).

To further investigate antioxidant properties of TUDCA, the measurements of oxygen uptake induced by Fenton reaction have been performed using EPR. Liposomes containing 35 mol % of (16:0)(22:6)PC, the lipid highly susceptible to oxidation, were exposed to free radical flux generated in the Fenton reaction in the presence and absence of TUDCA or UDCA. The rate of oxygen uptake (dO_2_/dt) in liposomes containing none of bile acids studied (control liposomes) was 9.02 ± 0.70 μM/min. In the presence of TUDCA, oxygen consumption rate was reduced by nearly 25% to 6.77 ± 0.83 μM/min (* *p* < 0.05) and the difference was statistically significant (Figure 3).

In case of UDCA enriched liposomes dO_2_/dt also seems to be slightly reduced (8.28 ± 0.08 μM/min), however this difference was not statistically significant. Oxygen uptake rate in samples, in which the Fenton reaction could not occur due to the lack of ferrous ions did not exceed 1.48 ± 1.21 µM/min (data not shown). Significant decrease in free radical-induced oxygen uptake in peroxidizable liposomes was observed only in the presence of TUDCA. UDCA was not as effective, although its ability to scavenge superoxide anion and hydroxyl radical has been demonstrated previously [26,27]. The key difference between these two bile acids is a taurine residue present in the TUDCA molecule. Although taurine antioxidant properties are rather controversial, it has been shown that it exerts an efficient reactivity towards nitric oxide, superoxide anion and prevents thiol groups against oxidation [67]. Taurine is also known for its ability to increase the level of iron-storing protein [68], activity of antioxidant enzymes [69] and to reduce lipid peroxidation [67,70]. Nevertheless, taurine alone has no impact of oxygen consumption rate in the system studied (data not shown). In 1990 DeLange and Glazer in their report suggested that the antioxidant activity of bile acids results from their ability to react with peroxyl radical [71]. In this reaction the 7α-hydroxyl group of the bile acid undergoes oxidation and its 7-keto derivative is formed [71]. In our system free radical flux is generated rather in the water phase, and TUDCA, which localizes in the interphase region of liposomes (as it is discussed later) may be the first substrate for superoxide anion or hydroxyl radical generated in this reaction rather than for peroxyl radical, which may be generated in the lipid bilayer. Recently, it has been shown that TUDCA alleviated H_2_O_2_-induced oxidative stress in neonatal rat cardiomyocytes [72] and ARPE-19 cells [25]. According to some reports, at least primary bile acids (i.e., taurocholic and glycocholic) are able to chelate iron and to form stable Fe^3+^-complexes [73]. Unfortunately, such unambiguous data are missing in the case of TUDCA. However, the mechanism of action of TUDCA in cells is much more complex than in the liposomal system, in which rather direct reaction of bile acid with reactive oxygen species should be considered as predominant.

### 3.3. Location of TUDCA in the Membrane—Molecular Dynamics Simulation

**Figure 4:**
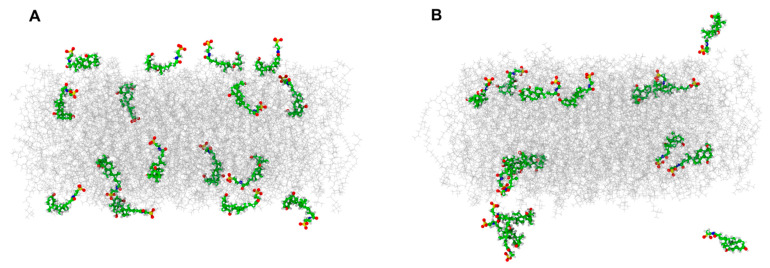
MD simulation of TUDCA in the POPC membrane. (**A**)—start, 0 ns, (**B**)—end, 500 ns. Colors of atoms: red—oxygen, yellow—sulphur, blue—nitrogen and green—carbon. Water molecules are removed for clarity. Animation showing TUDCA molecules movement is provided as supplementary material (Animation S1).

Figure 4 shows the location of TUDCA in POPC bilayer as a result of 500 ns MD simulation. The starting conditions (Figure 4A) were as follows: 8 TUDCA molecules lying horizontally on the membrane and 8 TUDCA molecules placed within the membrane perpendicular to the membrane surface (among them four with their sulphate group directed towards the membrane surface and four with their sulphate group directed towards the membrane center). After 500 ns of system equilibration some of the originally horizontally placed molecules remained in the water phase and three of them aggregated, other immersed themselves in the membrane remaining however close to the surface and adopting mostly a horizontal position, while most of those originally placed perpendicularly adopted a more horizontal position (Figure 4B). In every case sulphate groups of TUDCA molecules got directed towards the water phase. Z–coordinates plots for the movements of each of the eight TUDCA molecules during the entire time of MD simulations are presented in Figure S1 in the supplementary material.

To get a more detailed picture of TUDCA behavior in the membrane, the number of hydrogen bonds between different groups of TUDCA and POPC and water was calculated. The results ae presented in Table 2.

The sulphate group of TUDCA forms about five times more hydrogen bonds with water compared to hydroxyl groups (OH3 and OH7) and carbonyl oxygen (OE1) and the amine group of TUDCA forms about five times less hydrogen bonds with water compared to hydroxyl groups. This means that TUDCA molecules lie horizontally at the inner side of the interphase. Radial distribution functions (RDFs) of water oxygen atoms relative to TUDCA oxygen atoms are presented in the supplementary material (Figure S2). The amide group practically does not form hydrogen bonds with POPC, OH3 group interacts mostly with carbonyl oxygen of POPC, and OH7 group forms hydrogen bonds with oxygens of phosphate and carbonyl groups. This may be caused by the bending of sterol ring of TUDCA.

To make the overall picture of TUDCA localization in the POPC membrane much clearer, mass density profiles along the bilayer normal for the atoms of water, POPC, phosphorous (P), and TUDCA have been provided as a Figure S3 in the Supplementary material.

### 3.4. Distribution of TUDCA between DRM and DSM Domains

**Figure 5:**
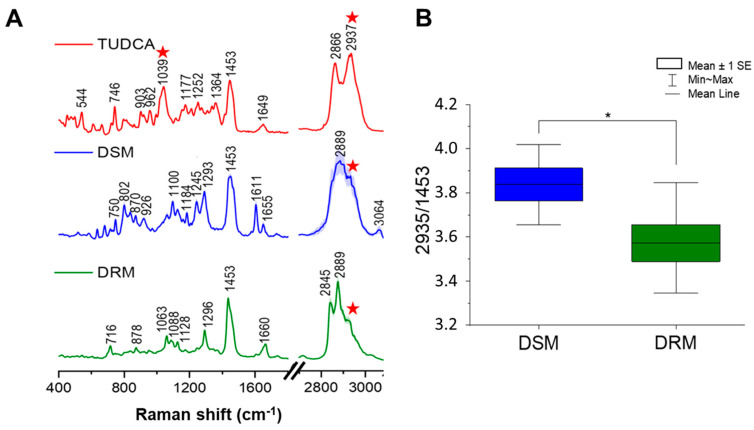
(**A**) The comparison of the average Raman spectra of TUDCA standard (red) with average spectra of DSM (blue) and DRM (green) where the SD is presented as shadow. All intensities of the bands presented in the region of 400–1800 cm^−1^ were tripled comparing to the 2800–3100 cm^−1^ region in order to visualize low intensity bands of the fingerprint region. The bands at 1039 cm^−1^ and 2937 cm^−1^ or only at 2937 cm^−1^, characteristic of TUDCA, are marked with a red star in the Raman spectrum of TUDCA and DRM and DSM spectra, respectively. (**B**) The comparison of band ratio of 2937/1453 calculated for DSM and DRM providing the semiquantitative information about a higher concentration of TUDCA inside measured samples. Results are given as mean ± SE, data distribution is presented as box plots (mean value, mean ± SE, min–max whiskers). Data normality was assessed using the Shapiro–Wilk test. The significance of the differences between the band ratio for DSM and DRM was evaluated by a one–way ANOVA with Tukey’s test (* *p* < 0.05).

Another interesting aspect of the TUDCA location in the membrane, especially taking into consideration the POS membranes, is its preference for the liquid-disordered phase [74]. Membranes of POS have very characteristic lipid composition. Nearly equimolar concentrations of unsaturated fatty acids, saturated fatty acids and cholesterol make them very similar to the raft-forming mixtures. Therefore, raft domains (in the form of detergent-resistant membranes DRM) can be isolated from the photoreceptor membranes by the cold Triton X-100 extraction [75]. After extracting DRM and DSM fractions from our raft-forming mixtures (see p. 2,7) we performed the analysis of TUDCA distribution between these fractions using Raman spectroscopy. RS is a technique widely used in lipids studies which is applied not only to analysis of lipid standards [76] but also for analysis of lipid fractions in cells [77,78,79,80] and tissues [81,82] including the semiquantitative approach. Figure 5 presents the comparison of the Raman spectrum of TUDCA standard compound (red) and the average Raman spectra of DSM (blue) and DRM (green) with the spectra standard deviation presented as shadow. Average spectra were obtained from ten single spectra acquired from the different, randomly selected areas of samples as described in the Materials and Methods section.

The analysis of the Raman spectrum of DRM suggests that the sample contains mainly saturated lipids including sphingomyelin (SM) and cholesterol groups. As previously reported, the RS technique can easily provide the information about the general lipid unsaturation level, for example, based on the RS intensity ratio of the bands located at around 1266/1300 cm^−1^ [81]. In the case of the Raman spectrum of DRM the band at around 1266 cm^–1^ [81], which is originated from the bending vibration of =CH group, is almost invisible, which indicates the minority of unsaturated lipids in this sample. Moreover, the information about lipid saturation can be delivered by the analysis of the H–C=/CH_2_ or CH_3_ groups. Bands at 2881 cm^–1^ and 2974 cm^−1^ were previously assigned predominantly to C–H stretches in CH_3_ groups, while bands at 3013 cm^−1^ and 3064 cm^−1^ were previously found to be associated with C–H stretch in H–C=C groups [83]. The analysis of observed bands for DRM suggests the presence of sphingomyelin, which was previously reported [82,84], has characteristic bands at around 720 cm^−1^, 1296 cm^−1^ and the characteristic triplet of bands at 1063, 1088 and 1129 cm^−1^. On the other hand, the presence of the shoulder at around 705 cm^−1^ and bands at 1660 and 1735 cm^−1^ suggests the presence of a cholesterol moiety [81,82,85]. The analysis of the C–H stretching region, with the strongest bands at 2852 and 2889 cm^−1^ confirms the presence of the mixture of SM and cholesterol.

Contrary to DRM, the DSM contains visible bands at around 1245 and 1655 cm^−1^, originated from the bending vibration of =CH group and stretching ν(C=C) modes, respectively, which suggests the presence of unsaturated lipids in this sample. Moreover, the presence of band at 3064 cm^−1^ confirms the presence of C–H stretch in H–C=C groups [83]. The presence of bands at around 750 cm^−1^, 870 cm^−1^, broad band at 1100 cm^−1^ and bands at 1611 and 1655 cm^−1^ suggests the majority of phosphatidylcholine (PC) moiety [82]. The CH stretching region with the maximum intensity at around 2889 cm^−1^ also suggests the presence of PC and the unsaturated lipids [76,82].

It was previously reported that the main bands for taurine in the solid state are observed at around 530, 735 and 1050 cm^−1^, which correspond to the deformation vibration of SO_3_, stretching modes of C–S and C–N groups, respectively [86,87]. On the other hand, the Raman spectrum of taurine in solution was previously reported to show a main strong band at 1045 cm^−1^ corresponding vibrations to the SO_3_ group [86]. The Raman spectrum of TUDCA revealed some characteristic bands, including the strongest band of the fingerprint region at 1039 cm^−1^ related to the presence of a taurine moiety. Unfortunately, although the band at 1039 cm^−1^ was the strongest marker band of TUDCA in the fingerprint region, its intensity was very low in the measured sample of DSM and was invisible in DRM as it overlapped with other bands in this range. Therefore, the semiquantitative analysis cannot be performed on the basis of the 1039 cm^−1^ band. In order to carry out the semiquantitative analysis of the presence of TUDCA in measured samples we focused on the analysis of the most intensive Raman bands in the region of 2800–3050 cm^−1^. As explained above, such a region differs between DRM and DSM, and TUDCA has also a distinct shape of such a region including the intensive band at 2937 cm^−1^. In order to determine which of these samples has a higher concentration of TUDCA we calculated the ratio of the relative integral intensity of the band at 2937 cm^−1^ to the relative integral intensity of the band at 1453 cm^−1^. The band at 1453 cm^−1^ originated from the C–H bending modes, which constitute the similar contribution in both samples. As presented in Figure 5B, a band ratio of 2937/1453 cm^−1^ for DSM and DRM provides the semiquantitative level of TUDCA inside measured samples and shows statistically significant higher level of TUDCA in DSM than in DRM. This confirms that TUDCA preferentially accumulates in the fraction rich in unsaturated lipids.

Similar preference for liquid-disordered phase rich in unsaturated lipids in the case of a model of POS membranes was observed for macular xanthophylls lutein and zeaxanthin, which are known antioxidants protecting retina against oxidative stress [88,89]. It was hypothesized that accumulation of xanthophylls in the region rich in unsaturated lipids is an additional mechanism enhancing their antioxidant action [90,91]. It seems possible that also TUDCA by accumulating in the liquid-disordered phase of POS membranes can more clearly manifest its antioxidant properties against free-radicals. Moreover, location of TUDCA in the liquid-disordered domain may facilitate its interacting with Rh, which is also predominantly present in this domain [92]. It has been shown that TUDCA interacts specifically with Rh stabilizing its active form, metarhodopsin II [93].

### 3.5. Effect of TUDCA on Membrane Structural Properties

#### 3.5.1. Polarity Profiles

**Figure 6:**
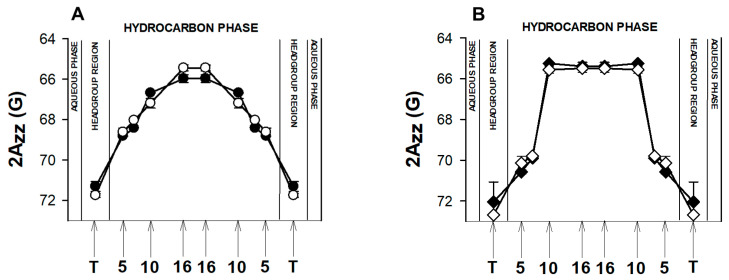
Polarity profiles across POPC/DMPE membranes containing 5 (**A**) or 40 (**B**) mol% cholesterol. The spectra were acquired at 120 K. Upward changes of 2Azz indicate a decrease in polarity. Full symbols denote a control membrane and empty symbols—a membrane containing 5 mol% TUDCA. Approximate locations of the nitroxide moieties of spin labels are indicated by arrows.

2Azz, which is a z–component of the hyperfine interaction tensor of the nitroxide spin label, can be measured directly from the EPR spectra of immobilized spin labels (such as in frozen liposome samples) as a distance between the outermost spectral extrema [94]. This parameter reflects the polarity of local environment of a nitroxide moiety [95]. Higher values of 2Azz indicate higher polarity (lower hydrophobicity) and polarity profiles across the lipid bilayer reflect water penetration into the membrane [95]. This can be affected by lipid composition, presence of cholesterol, carotenoids, peptides, or drugs [94,96,97,98,99]. Here, we analyzed the effect of TUDCA on polarity profiles across two simple models of membranes containing high and low level of cholesterol (Figure 6).

Both polarity profiles presented in Figure 6 are typical for membranes composed of unsaturated lipids (here POPC) whose hydrophobicity level in the central part is rather high [94]. However, the shape of the profiles is different in these two models: the hydrophobicity of the membrane containing 5 mol % cholesterol increased gradually toward the membrane center whereas the presence of 40 mol % cholesterol made the shape of the hydrophobicity barrier rectangular with a sharp change in 2Azz values between the 7th and 10th carbon. Such differences between the polarity profiles of membranes mimicking the membranes of young and old disks of POS were observed previously [98]. The effect of TUDCA on polarity profiles of both membranes was not significant. A slight effect of increased polarity can be seen in the polar headgroup region, which may be caused by the presence of polar sulphate, hydroxyl and amide groups of TUDCA in this region. As described above, our MD simulation data show that TUDCA molecules locate in the membrane mostly parallel to the surface with their sulphate groups directed towards the water phase and forming hydrogen bonds with water, and other polar groups interacting with carbonyl and phosphate groups of lipids via hydrogen bonds (Figure 4). Additionally, according to the literature, TUDCA does not penetrate membranes deeply, but adsorbs to the lipid/water interphase [74].

#### 3.5.2. Order Parameter

**Figure 7:**
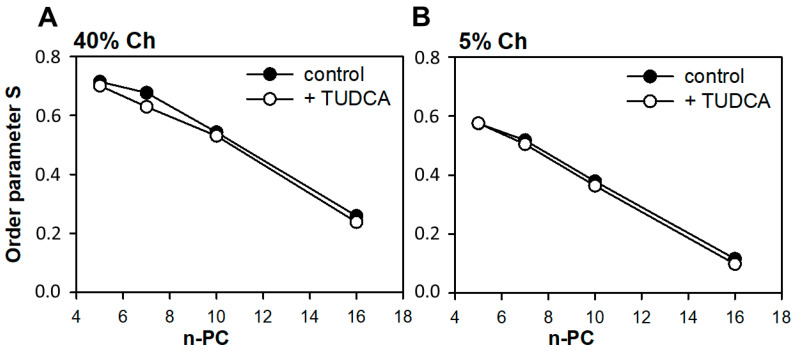
Order parameter of 5–, 7–, 10– and 16–PC in POPC/DMPE membranes containing 40 (**A**) or 5 (**B**) mol% cholesterol. Spectra were acquired at 310 K. Full symbols denote a control membrane and empty symbols—a membrane containing 5 mol % TUDCA.

To study the effect of TUDCA on the order of lipid acyl chains in both simple models of POS membranes, the order parameter S was calculated based on the EPR spectra of 5–, 7–, 10– and 16–PC spin labels according to Marsh [100]. Additionally, MD simulations were used to obtain the molecular order parameter S_mol_ for the POPC membrane [48]. In the case of n–PC, S reflects the segmental order parameter of the hydrocarbon chain segment to which the nitroxide fragment is attached. The values of S parameter are summed up in Figure 7.

As expected, in both membranes the values of S parameter decreased in the direction from the region close to the membrane surface toward the membrane center. Additionally, the difference can be seen between both membranes, which results from different cholesterol content. The values of the S parameter were higher in the membrane containing 40 mol% cholesterol. This effect is observed at all positions along the acyl chain and reflects a well known rigidifying effect of cholesterol on membranes [101,102]. The effect of TUDCA on lipid order was not significant. However, the S values for TUDCA enriched membranes were slightly lower than for control membranes. In the case of the 7th carbon a difference was visible, since the presence of TUDCA decreased the value of S parameter by about 7%. For the low–cholesterol model the effect of TUDCA was even weaker, especially at the upper part of the acyl chain (5–PC) where no difference was observed whatsoever. In deeper parts of the membrane the values of S parameter in the presence of TUDCA were lower than in its absence by about 4% at (10–PC) and by about 15% (16–PC).

**Figure 8:**
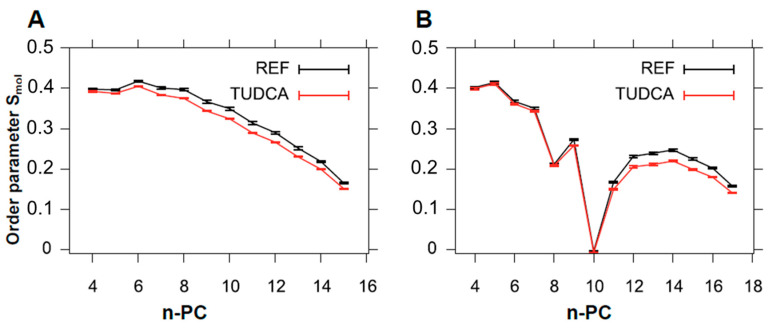
Molecular order parameter (S_mol_) calculated for POPC alone (REF) and in the presence of TUDCA (TUDCA) plotted against the carbon number in the alkyl chain. S_mol_ parameter was calculated for palmitic acid chain esterified in sn1 position (**A**) and for oleic acid chain esterified in the sn2 position (**B**) separately.

The values of the S_mol_ parameter calculated based on MD simulations are presented in Figure 8. In this case, S_mol_ was calculated separately for palmitic and oleic chains of POPC (positions sn1 and sn2, respectively). In both cases TUDCA slightly decreased the values of S_mol_ but the effect was stronger for the saturated chain. In the oleic chain the decrease of lipid order was noticeable only for the fragment below the 10^th^ carbon atom, which corresponded to the position of a double bond (C9–C10). Generally, the S_mol_ values were lower than the S values obtained by EPR, which could be explained by the fact that MD simulations were performed for a membrane without cholesterol. Nevertheless, the results obtained by both techniques were in agreement showing slight fluidizing effect of TUDCA on membranes, especially in their central part.

Increase in polarity and fluidity of membranes rich in cholesterol caused by the presence of TUDCA at its critical micelle concentration (CMC) has been reported previously [103]. However, Arai et al. have hypothesized that TUDCA penetrates into the cell membrane, where it induces the release of cholesterol. Our data show that the effect of TUDCA on structural properties of POS model membranes is weak and does not depend on cholesterol content. It is generally in agreement with previously obtained data, however it was shown previously that especially cholesterol enriched membranes do not have affinity to bile acids [74]. Although we did not see the difference in the TUDCA effect on membranes depending on cholesterol content, we did observe that TUDCA only slightly penetrates into cholesterol-rich membrane domains (Figure 5).

## 4. Conclusions

The main goal of our study was to analyze whether the protective effect of TUDCA against photoreceptor photodamage could be facilitated by its effects on membrane structural properties. The results of MD simulations show that TUDCA molecules did not penetrate deeply the lipid bilayer of model membranes but located mostly parallel to the membrane surface with their sulphate groups directed towards the water phase. This may explain rather weak effects of TUDCA on membrane properties such as lipid order and polarity observed by EPR spin labelling technique. However, this location may be beneficial for TUDCA’s antioxidant action against water soluble free radicals. Such an activity was demonstrated in our experiments with oxygen consumption in liposomes containing highly unsaturated lipids. Although no significant differences were observed in TUDCA’s effects on membranes with different cholesterol content, cholesterol presence may play a role in the antioxidant activity of TUDCA in the context of membrane domain structure. Our results obtained with Raman spectroscopy suggest that in domain-forming membranes composed of saturated phospholipids, unsaturated phospholipids and cholesterol, TUDCA partitions preferentially into detergent soluble domains, which are rich in unsaturated lipids while in detergent-resistant fraction, which is enriched in cholesterol, it is present in a smaller amount. By accumulating in the domain containing lipids susceptible to peroxidation, TUDCA can more clearly manifest its antioxidant properties against free radicals.

## Figures and Tables

**Table 1:** Summarized bimolecular rate constants of interaction of TUDCA and UDCA with singlet oxygen calculated from straight-line slopes presented in Figure 2B. Rate constant of ^1^O_2_ (^1^Δ_g_) quenching by taurine from Egorov et al. [64].

Compound	Rate constants for Interactions of ^1^O_2_ (M^−1^s^−1^)	Solvent
TUDCA	(1.98 ± 0.32) × 10^5^	DMSO-d_6_:CHCl_3_ mixture (1:1, v/v)
UDCA	(0.39 ± 0.13) × 10^5^	DMSO-d_6_:CHCl_3_ mixture (1:1, v/v)
Taurine ^a^	<3.00 × 10^3^	Deuterated water (D_2_O)
Phospholipids ^b^	0.8 ÷ 1.8 × 10^5^	Carbon tetrachloride (CCl_4_)
Cholesterol ^c^	5.7 ×·10^4^	Benzene
a–Tocopherol ^d^	(1.21 ± 0.17) × 10^8^	Carbon tetrachloride (CCl_4_)

^a^ Egorov, S., et al.; *Biochem. Mol. Biol. Int.* **1997**, *41*, 687–694; ^b^ Broniec, A., et al.; *Free Rad. Biol. Med.* **2011**, *50*, 892–898; ^c^ Vever-Bizet, C., et al.; *Photochem. Photobiol.* **1989**, *50*, 321–325; **^d^** Gruszka, J., et al.; *Free Rad. Biol. Med*. **2008**, *45*, 920–928.

**Table 2:** Average (by time and number of TUDCA molecules) number of hydrogen bonds between TUDCA (considering single atoms) and POPC (considering oxygen atoms of phosphate and carbonyl groups) and between TUDCA and water. POPC: OP–phosphate group, OC–carbonyl group, TUDCA: OS–sulphate group, NH–amide group, OE1–carbonyl group, OH3 and OH7–hydroxyl groups at the 3rd and the 7th carbon atom of a sterol ring, respectively. Only TUDCA molecules which are present in the membrane are considered.

	TUDCA Molecule				
	Sulphate Group OS	Amide Group NH	Carbonyl Group OE1	Hydroxyl Group at the 3rd Carbon Atom of the Sterol Ring OH3	Hydroxyl Group at the 7th Carbon Atom of the Sterol Ring OH7
POPC	—	0.03 ± 0.05	*—*	0.4 ± 0.14	0.35 ± 0.14
Phosphate group OP	—	0.01 ± 0.03	—	0.07 ± 0.08	0.15 ± 0.1
Carbonyl group OC	—	0.03 ± 0.05	—	0.33 ± 0.14	0.19 ± 0.13
WATER	4.93 ± 0.42	0.19 ± 0.12	0.87 ± 0.19	1.02 ± 0.28	0.98 ± 0.22

## Data Availability

Data supporting reported results can be found here: https://ruj.uj.edu.pl/xmlui/handle/item/266783, accessed on 8 March 2021.

## Supplementary Materials

The following are available online at https://www.mdpi.com/article/10.3390/membranes11050327/s1, Figure S1. Z-coordinate plots for the center of mass (black line), sulfur (yellow) and O3 oxygen (red) atoms of four horizontally located molecules of TUDCA, which remained in a water phase for the entire simulation time. Dashed gray lines indicate the mean (from the entire simulation time) position of the P (phosphorus) atom of lipids; Figure S2. Z-coordinate plots for the center of mass (black line), sulfur (yellow) and O3 oxygen (red) atoms of four horizontally arranged molecules of TUDCA, which incorporated into the POPC membrane at different times of simulations. Dashed gray lines indicate the mean (from the entire simulation time) position of the P (phosphorus) atom of lipids; Figure S3. Z–coordinate plots for the center of mass (black line), sulfur (yellow) and O3 oxygen (red) atoms of four vertically located TUDCA molecules with sulphate group directed into the water phase. These molecules localized in the interphase region of POPC membrane. Dashed gray lines indicate the mean (from the entire simulation time) position of the P (phosphorus) atom of lipids; Figure S4. Radial distribution functions (RDFs) of water oxygen atoms relative to TUDCA oxygen atoms: O1S, O2S, O3S—oxygen atoms in the sulphate group, OE1—oxygen in the carbonyl group and O3 and O7—oxygen in the 3rd and 7th hydroxyl groups. Stronger ordering of water by the oxygens present in the sulphate group than these of the hydroxyl groups is evident; Figure S5. Mass density profiles along the bilayer normal (z axis, bilayer center at 0) of the atoms of water, lipids (POPC), phosphorous (P) and TUDCA (all 16 molecules), VideoS1 Animation showing TUDCA molecules movement.

## References

[1] Hanafi N.I., Mohamed A.S., Sheikh Abdul Kadir S.H., Othman M.H.D. (2018). Overview of Bile Acids Signaling and Perspective on the Signal of Ursodeoxycholic Acid, the Most Hydrophilic Bile Acid, in the Heart. Biomolecules.

[2] Berger E., Haller D. (2011). Structure-function analysis of the tertiary bile acid TUDCA for the resolution of endoplasmic reticulum stress in intestinal epithelial cells. Biochem. Biophys. Res. Commun..

[3] Hagey L.R., Crombie D.L., Espinosa E., Carey M.C., Igimi H., Hofmann A.F. (1993). Ursodeoxycholic acid in the Ursidae: Biliary bile acids of bears, pandas, and related carnivores. J. Lipid Res..

[4] Solá S., Garshelis D.L., Amaral J.D., Noyce K.V., Coy P.L., Steer C.J., Iaizzo P.A., Rodrigues C.M.P. (2006). Plasma levels of ursodeoxycholic acid in black bears, Ursus americanus: Seasonal changes. Comp. Biochem. Physiol. Part C Toxicol. Pharmacol..

[5] Vang S., Longley K., Steer C.J., Low W.C. (2014). The Unexpected Uses of Urso- and Tauroursodeoxycholic Acid in the Treatment of Non-liver Diseases. Glob. Adv. Health Med..

[6] Ramalho R.M., Viana R.J., Low W.C., Steer C.J., Rodrigues C.M. (2008). Bile acids and apoptosis modulation: An emerging role in experimental Alzheimer’s disease. Trend. Mol. Med..

[7] Keene C.D., Rodrigues C.M., Eich T., Chhabra M.S., Steer C.J., Low W.C. (2002). Tauroursodeoxycholic acid, a bile acid, is neuroprotective in a transgenic animal model of Huntington’s disease. Proc. Natl. Acad. Sci. USA.

[8] Grant S.M., DeMorrow S. (2020). Bile Acid Signaling in Neurodegenerative and Neurological Disorders. Int. J. Mol. Sci..

[9] Boatright J.H., Moring A.G., McElroy C., Phillips M.J., Do V.T., Chang B., Hawes N.L., Boyd A.P., Sidney S.S., Stewart R.E. (2006). Tool from ancient pharmacopoeia prevents vision loss. Mol. Vis..

[10] Feng Y., Siu K., Wang N., Ng K.-M., Tsao S.-W., Nagamatsu T., Tong Y. (2009). Bear bile: Dilemma of traditional medicinal use and animal protection. J. Ethnobiol. Ethnomed..

[11] Ludolph A.C. (2016). The TUDCA trial—Innovative trial designs for amyotrophic lateral sclerosis drugs?. Eur. J. Neurol..

[12] Castro-Caldas M., Carvalho A.N., Rodrigues E., Henderson C.J., Wolf C.R., Rodrigues C.M., Gama M.J. (2012). Tauroursodeoxycholic acid prevents MPTP-induced dopaminergic cell death in a mouse model of Parkinson’s disease. Mol. Neurobiol..

[13] Rodrigues C.M., Stieers C.L., Keene C.D., Ma X., Kren B.T., Low W.C., Steer C.J. (2000). Tauroursodeoxycholic acid partially prevents apoptosis induced by 3-nitropropionic acid: Evidence for a mitochondrial pathway independent of the permeability transition. J. Neurochem..

[14] Oveson B.C., Iwase T., Hackett S.F., Lee S.Y., Usui S., Sedlak T.W., Snyder S.H., Campochiaro P.A., Sung J.U. (2011). Constituents of bile, bilirubin and TUDCA, protect against oxidative stress-induced retinal degeneration. J. Neurochem..

[15] Drack A.V., Dumitrescu A.V., Bhattarai S., Gratie D., Stone E.M., Mullins R., Sheffield V.C. (2012). TUDCA slows retinal degeneration in two different mouse models of retinitis pigmentosa and prevents obesity in Bardet-Biedl syndrome type 1 mice. Invest. Ophthalmol. Vis. Sci..

[16] Zhang T., Baehr W., Fu Y. (2012). Chemical chaperone TUDCA preserves cone photoreceptors in a mouse model of Leber congenital amaurosis. Invest. Ophthalmol. Vis. Sci..

[17] Phillips M.J., Walker T.A., Choi H.Y., Faulkner A.E., Kim M.K., Sidney S.S., Boyd A.P., Nickerson J.M., Boatright J.H., Pardue M.T. (2008). Tauroursodeoxycholic acid preservation of photoreceptor structure and function in the rd10 mouse through postnatal day 30. Invest. Ophthalmol. Vis. Sci..

[18] Amaral J.D., Viana R.J., Ramalho R.M., Steer C.J., Rodrigues C.M. (2009). Bile acids: Regulation of apoptosis by ursodeoxycholic acid. J. Lipid Res..

[19] Uppala J.K., Gani A.R., Ramaiah K.V.A. (2017). Chemical chaperone, TUDCA unlike PBA, mitigates protein aggregation efficiently and resists ER and non-ER stress induced HepG2 cell death. Sci. Rep..

[20] Xie Q., Khaoustov V.I., Chung C.C., Sohn J., Krishnan B., Lewis D.E., Yoffe B. (2002). Effect of tauroursodeoxycholic acid on endoplasmic reticulum stress-induced caspase-12 activation. Hepatology.

[21] Fonseca I., Gordino G., Moreira S., Nunes M.J., Azevedo C., Gama M.J., Rodrigues E., Rodrigues C.M.P., Castro-Caldas M. (2017). Tauroursodeoxycholic Acid Protects Against Mitochondrial Dysfunction and Cell Death via Mitophagy in Human Neuroblastoma Cells. Mol. Neurobiol..

[22] Rodrigues C.M., Sola S., Brito M.A., Brondino C.D., Brites D., Moura J.J. (2001). Amyloid beta-peptide disrupts mitochondrial membrane lipid and protein structure: Protective role of tauroursodeoxycholate. Biochem. Biophys. Res. Commun..

[23] Peng S., Huo X., Rezaei D., Zhang Q., Zhang X., Yu C., Asanuma K., Cheng E., Pham T.H., Wang D.H. (2014). In Barrett’s esophagus patients and Barrett’s cell lines, ursodeoxycholic acid increases antioxidant expression and prevents DNA damage by bile acids. Am. J. Physiol. Gastrointest. Liver Physiol..

[24] Moreira S., Fonseca I., Nunes M.J., Rosa A., Lemos L., Rodrigues E., Carvalho A.N., Outeiro T.F., Rodrigues C.M.P., Gama M.J. (2017). Nrf2 activation by tauroursodeoxycholic acid in experimental models of Parkinson’s disease. Exp. Neurol..

[25] Alhasani R.H., Almarhoun M., Zhou X., Reilly J., Patterson S., Zeng Z., Shu X. (2020). Tauroursodeoxycholic Acid Protects Retinal Pigment Epithelial Cells from Oxidative Injury and Endoplasmic Reticulum Stress In Vitro. Biomedicines.

[26] Ljubuncic P., Abu-Salach O., Bomzon A. (2005). Ursodeoxycholic acid and superoxide anion. World J. Gastroenterol..

[27] Lapenna D., Ciofani G., Festi D., Neri M., Pierdomenico S.D., Giamberardino M.A., Cuccurullo F. (2002). Antioxidant properties of ursodeoxycholic acid. Biochem. Pharmacol..

[28] Heuman D.M., Bajaj R. (1994). Ursodeoxycholate conjugates protect against disruption of cholesterol-rich membranes by bile salts. Gastroenterology.

[29] Burns M.E., Arshavsky V.Y. (2005). Beyond counting photons: Trials and trends in vertebrate visual transduction. Neuron.

[30] Ding J.-D., Salinas R.Y., Arshavsky V.Y. (2015). Discs of mammalian rod photoreceptors form through the membrane evagination mechanism. J. Cell Biol..

[31] Boesze-Battaglia K., Hennessey T., Albert A.D. (1989). Cholesterol heterogeneity in bovine rod outer segment disk membranes. J. Biol. Chem..

[32] Boesze-Battaglia K., Schimmel R. (1997). Cell membrane lipid composition and distribution: Implications for cell function and lessons learned from photoreceptors and platelets. J. Exp. Biol..

[33] Redmond R.W., Gamlin J.N. (1999). A compilation of singlet oxygen yields from biologically relevant molecules. Photochem. Photobiol..

[34] Bonnett R., McGarvey D.J., Harriman A., Land E.J., Truscott T.G., Winfield U.J. (1988). Photophysical properties of meso-tetraphenylporphyrin and some meso-tetra(hydroxyphenyl)porphyrins. Photochem. Photobiol..

[35] Rozanowska M., Jarvis-Evans J., Korytowski W., Boulton M.E., Burke J.M., Sarna T. (1995). Blue light-induced reactivity of retinal age pigment. In vitro generation of oxygen-reactive species. J. Biol. Chem..

[36] Zadlo A., Burke J.M., Sarna T. (2009). Effect of untreated and photobleached bovine RPE melanosomes on the photoinduced peroxidation of lipids. Photochem. Photobiol. Sci..

[37] DeLano W.L. (2002). Pymol an open–source molecular graphics tool. CCP4 Newsl. Protein Crystallogr..

[38] Jo S., Kim T., Iyer V.G., Im W. (2008). CHARMM-GUI: A web-based graphical user interface for CHARMM. J. Comput. Chem..

[39] Huang J., MacKerell A.D. (2013). CHARMM36 all-atom additive protein force field: Validation based on comparison to NMR data. J. Comput. Chem..

[40] Vanommeslaeghe K., Hatcher E., Acharya C., Kundu S., Zhong S., Shim J., Darian E., Guvench O., Lopes P., Vorobyov I. (2010). CHARMM general force field: A force field for drug-like molecules compatible with the CHARMM all-atom additive biological force fields. J. Comput. Chem..

[41] Jorgensen W.L., Chandrasekhar J., Madura J.D., Impey R.W., Klein M.L. (1983). Comparison of simple potential functions for simulating liquid water. J. Chem. Phys..

[42] Essmann U., Perera L., Berkowitz M.L., Darden T., Lee H., Pedersen L.G. (1995). A smooth particle mesh Ewald method. J. Chem. Phys..

[43] Seelig J., Waespe-Sarcevic N. (1978). Molecular order in cis and trans unsaturated phospholipid bilayers. Biochemistry.

[44] Nosé S. (1984). A unified formulation of the constant temperature molecular dynamics methods. J. Chem. Phys..

[45] Hoover W.G. (1985). Canonical dynamics: Equilibrium phase-space distributions. Phys. Rev. A Gen. Phys..

[46] Parrinello M., Rahman A. (1981). Polymorphic transitions in single crystals: A new molecular dynamics method. J. Appl. Phys..

[47] Markiewicz M., Pasenkiewicz-Gierula M. (2011). Comparative Model Studies of Gastric Toxicity of Nonsteroidal Anti-Inflammatory Drugs. Langmuir.

[48] Pasenkiewicz-Gierula M., Róg T., Grochowski J., Serda P., Czarnecki R., Librowski T., Lochyński S. (2003). Effects of a Carane Derivative Local Anesthetic on a Phospholipid Bilayer Studied by Molecular Dynamics Simulation. Biophys. J..

[49] Gandhavadi M., Allende D., Vidal A., Simon S.A., McIntosh T.J. (2002). Structure, composition, and peptide binding properties of detergent soluble bilayers and detergent resistant rafts. Biophys. J..

[50] Folch J., Lees M., Sloane Stanley G.H. (1957). A simple method for the isolation and purification of total lipides from animal tissues. J. Biol. Chem..

[51] Ogilby P.R. (2010). Singlet oxygen: There is indeed something new under the sun. Chem. Soc. Rev..

[52] Schmidt R. (2006). Photosensitized generation of singlet oxygen. Photochem. Photobiol..

[53] Krasnovsky A.A., Kagan V.E. (1979). Photosensitization and quenching of singlet oxygen by pigments and lipids of photoreceptor cells of the retina. FEBS Lett..

[54] Rozanowska M., Sarna T. (2005). Light-induced damage to the retina: Role of rhodopsin chromophore revisited. Photochem. Photobiol..

[55] Yu D.Y., Cringle S.J., Su E.N. (2005). Intraretinal oxygen distribution in the monkey retina and the response to systemic hyperoxia. Invest. Ophthalmol. Vis. Sci..

[56] Hao W., Wenzel A., Obin M.S., Chen C.K., Brill E., Krasnoperova N.V., Eversole-Cire P., Kleyner Y., Taylor A., Simon M.I. (2002). Evidence for two apoptotic pathways in light-induced retinal degeneration. Nat. Genet..

[57] Boulton M., Rózanowska M., Rózanowski B. (2001). Retinal photodamage. J. Photochem. Photobiol. B.

[58] Maeda T., Golczak M., Maeda A. (2012). Retinal photodamage mediated by all-trans-retinal. Photochem. Photobiol..

[59] Saari J.C., Garwin G.G., van Hooser J.P., Palczewski K. (1998). Reduction of all-trans-retinal limits regeneration of visual pigment in mice. Vision Res..

[60] Harper W.S., Gaillard E.R. (2001). Studies of all-trans-retinal as a photooxidizing agent. Photochem. Photobiol..

[61] Rozanowska M., Wessels J., Boulton M., Burke J.M., Rodgers M.A., Truscott T.G., Sarna T. (1998). Blue light-induced singlet oxygen generation by retinal lipofuscin in non-polar media. Free Radic. Biol. Med..

[62] Acar N., Berdeaux O., Gregoire S., Cabaret S., Martine L., Gain P., Thuret G., Creuzot-Garcher C.P., Bron A.M., Bretillon L. (2012). Lipid composition of the human eye: Are red blood cells a good mirror of retinal and optic nerve fatty acids?. PLoS ONE.

[63] Koscielniak A., Serafin M., Duda M., Oles T., Zadlo A., Broniec A., Berdeaux O., Gregoire S., Bretillon L., Sarna T. (2017). Oxidation-Induced Increase In Photoreactivity of Bovine Retinal Lipid Extract. Cell Biophys..

[64] Egorov S., Kurella E.G., Boldyrev A.A., Krasnovsky A.A. (1997). Quenching of singlet molecular oxygen by carnosine and related antioxidants. Monitoring 1270-nm phosphorescence in aqueous media. Biochem. Mol. Biol. Int..

[65] Michaeli A., Feitelson J. (1994). Reactivity of singlet oxygen toward amino acids and peptides. Photochem. Photobiol..

[66] Broniec A., Klosinski R., Pawlak A., Wrona-Krol M., Thompson D., Sarna T. (2011). Interactions of plasmalogens and their diacyl analogs with singlet oxygen in selected model systems. Free Radic. Biol. Med..

[67] Oliveira M.W., Minotto J.B., de Oliveira M.R., Zanotto-Filho A., Behr G.A., Rocha R.F., Moreira J.C., Klamt F. (2010). Scavenging and antioxidant potential of physiological taurine concentrations against different reactive oxygen/nitrogen species. Pharmacol. Rep..

[68] Seidel U., Lüersen K., Huebbe P., Rimbach G. (2020). Taurine Enhances Iron-Related Proteins and Reduces Lipid Peroxidation in Differentiated C2C12 Myotubes. Antioxidants.

[69] Zhang Z., Liu D., Yi B., Liao Z., Tang L., Yin D., He M. (2014). Taurine supplementation reduces oxidative stress and protects the liver in an iron-overload murine model. Mol. Med. Rep..

[70] You J.S., Chang K.J. (1998). Effects of taurine supplementation on lipid peroxidation, blood glucose and blood lipid metabolism in streptozotocin-induced diabetic rats. Adv. Exp. Med. Biol..

[71] DeLange R.J., Glazer A.N. (1990). Bile acids: Antioxidants or enhancers of peroxidation depending on lipid concentration. Arch. Biochem. Biophys..

[72] Zhang L., Wang Y. (2018). Tauroursodeoxycholic Acid Alleviates H(_2_)O(_2_)-Induced Oxidative Stress and Apoptosis via Suppressing Endoplasmic Reticulum Stress in Neonatal Rat Cardiomyocytes. Dose Response Publ. Int. Horm. Soc..

[73] Urdaneta V., Casadesús J. (2017). Interactions between Bacteria and Bile Salts in the Gastrointestinal and Hepatobiliary Tracts. Front. Med..

[74] Mello-Vieira J., Sousa T., Coutinho A., Fedorov A., Lucas S.D., Moreira R., Castro R.E., Rodrigues C.M., Prieto M., Fernandes F. (2013). Cytotoxic bile acids, but not cytoprotective species, inhibit the ordering effect of cholesterol in model membranes at physiologically active concentrations. Biochim. Biophys. Acta.

[75] Elliott M.H., Nash Z.A., Takemori N., Fliesler S.J., McClellan M.E., Naash M.I. (2008). Differential distribution of proteins and lipids in detergent-resistant and detergent-soluble domains in rod outer segment plasma membranes and disks. J. Neurochem..

[76] Czamara K., Majzner K., Pacia M.Z., Kochan K., Kaczor A., Baranska M. (2015). Raman spectroscopy of lipids: A review. J. Raman Spectrosc..

[77] Blat A., Stepanenko T., Bulat K., Wajda A., Dybas J., Mohaissen T., Alcicek F.C., Szczesny-Malysiak E., Malek K., Fedorowicz A. (2021). Spectroscopic Signature of Red Blood Cells in a D-Galactose-Induced Accelerated Aging Model. Int. J. Mol. Sci..

[78] Dybas J., Bulat K., Blat A., Mohaissen T., Wajda A., Mardyla M., Kaczmarska M., Franczyk-Zarow M., Malek K., Chlopicki S. (2020). Age-related and atherosclerosis-related erythropathy in ApoE/LDLR(-/-) mice. Biochim. Biophys. Acta Mol. Basis Dis..

[79] Blat A., Dybas J., Kaczmarska M., Chrabaszcz K., Bulat K., Kostogrys R.B., Cernescu A., Malek K., Marzec K.M. (2019). An Analysis of Isolated and Intact RBC Membranes—A Comparison of a Semiquantitative Approach by Means of FTIR, Nano-FTIR, and Raman Spectroscopies. Anal. Chem..

[80] Heraud P., Marzec K.M., Zhang Q.H., Yuen W.S., Carroll J., Wood B.R. (2017). Label-free in vivo Raman microspectroscopic imaging of the macromolecular architecture of oocytes. Sci. Rep..

[81] Blat A., Dybas J., Chrabaszcz K., Bulat K., Jasztal A., Kaczmarska M., Pulyk R., Popiela T., Slowik A., Malek K. (2019). FTIR, Raman and AFM characterization of the clinically valid biochemical parameters of the thrombi in acute ischemic stroke. Sci. Rep..

[82] Kochan K., Chrabaszcz K., Szczur B., Maslak E., Dybas J., Marzec K.M. (2016). IR and Raman imaging of murine brains from control and ApoE/LDLR−/− mice with advanced atherosclerosis. Analyst.

[83] Jamieson L.E., Wetherill C., Faulds K., Graham D. (2018). Ratiometric Raman imaging reveals the new anti-cancer potential of lipid targeting drugs. Chem. Sci..

[84] Shirota K., Yagi K., Inaba T., Li P.-C., Murata M., Sugita Y., Kobayashi T. (2016). Detection of Sphingomyelin Clusters by Raman Spectroscopy. Biophys. J..

[85] Marzec K.M., Wrobel T.P., Rygula A., Maslak E., Jasztal A., Fedorowicz A., Chlopicki S., Baranska M. (2014). Visualization of the biochemical markers of atherosclerotic plaque with the use of Raman, IR and AFM. J. Biophotonics.

[86] Maiti N., Thomas S., Debnath A., Kapoor S. (2016). Raman and XPS study on the interaction of taurine with silver nanoparticles. RSC Adv..

[87] Lima R.J.C., Freire P.T.C., Sasaki J.M., Melo F.E.A., Mendes Filho J., Moreira R.L. (2001). Temperature-dependent Raman study of taurine single crystal. J. Raman Spectrosc..

[88] Wisniewska A., Subczynski W.K. (2006). Distribution of macular xanthophylls between domains in a model of photoreceptor outer segment membranes. Free Radic. Biol. Med..

[89] Wisniewska A., Subczynski W.K. (2006). Accumulation of macular xanthophylls in unsaturated membrane domains. Free Radic. Biol. Med..

[90] Wisniewska-Becker A., Nawrocki G., Duda M., Subczynski W.K. (2012). Structural aspects of the antioxidant activity of lutein in a model of photoreceptor membranes. Acta Biochim. Pol..

[91] Subczynski W., Wisniewska-Becker A., Widomska J., Landrum J.T., Nolan J.M. (2013). Xanthophyll–membrane interactions. Carotenoids and Retinal Disease.

[92] Polozova A., Litman B.J. (2000). Cholesterol dependent recruitment of di22:6-PC by a G protein-coupled receptor into lateral domains. Biophys. J..

[93] Lobysheva E., Taylor C.M., Marshall G.R., Kisselev O.G. (2018). Tauroursodeoxycholic acid binds to the G-protein site on light activated rhodopsin. Exp. Eye Res..

[94] Subczynski W.K., Wisniewska A., Yin J.-J., Hyde J.S., Kusumi A. (1994). Hydrophobic Barriers of Lipid Bilayer Membranes Formed by Reduction of Water Penetration by Alkyl Chain Unsaturation and Cholesterol. Biochemistry.

[95] Griffith O.H., Dehlinger P.J., Van S.P. (1974). Shape of the hydrophobic barrier of phospholipid bilayers (Evidence for water penetration in biological membranes). J. Membr. Biol..

[96] Wisniewska A., Subczynski W.K. (1998). Effects of polar carotenoids on the shape of the hydrophobic barrier of phospholipid bilayers. Biochim. Biophys. Acta Biomembr..

[97] Wisniewska A., Subczynski W.K. (1996). Spin-label study on gramicidin–phosphatidylcholine interface: Fluidity, hydrophobicity and ion penetration. Curr. Top. Biophys..

[98] Duda M., Kawula K., Pawlak A., Sarna T., Wisniewska-Becker A. (2017). EPR Studies on the Properties of Model Photoreceptor Membranes Made of Natural and Synthetic Lipids. Cell Biochem. Biophys..

[99] Duda M., Cygan K., Wisniewska-Becker A. (2020). Effects of Curcumin on Lipid Membranes: An EPR Spin-label Study. Cell Biochem. Biophys..

[100] Marsh D., Grell E. (1981). Electron spin resonance: Spin labels. Membrane Spectroscopy.

[101] Pasenkiewicz-Gierula M., Subczynski W.K., Kusumi A. (1990). Rotational diffusion of a steroid molecule in phosphatidylcholine-cholesterol membranes: Fluid-phase microimmiscibility in unsaturated phosphatidylcholine-cholesterol membranes. Biochemistry.

[102] Kusumi A., Subczynski W.K., Pasenkiewicz-Gierula M., Hyde J.S., Merkle H. (1986). Spin-label studies on phosphatidylcholine-cholesterol membranes: Effects of alkyl chain length and unsaturation in the fluid phase. Biochim. Biophys. Acta.

[103] Arai Y., Choi B., Kim B.J., Rim W., Park S., Park H., Ahn J., Lee S.-H. (2019). Tauroursodeoxycholic acid (TUDCA) counters osteoarthritis by regulating intracellular cholesterol levels and membrane fluidity of degenerated chondrocytes. Biomater. Sci..

