# Supplementary material for: Tauroursodeoxycholic Acid (TUDCA)—Lipid Interactions and Antioxidant Properties of TUDCA Studied in Model of Photoreceptor Membranes
Source: Membranes (Basel). 2021 Apr 29;11(5):327. doi: 10.3390/membranes11050327 (PMC8146903; doi:10.3390/membranes11050327)
Supplement: Supplementary file 1 [file membranes-11-00327-s001.zip › membranes-1156431-supplementary/resizemembranes-1156431 supplementary/Supplementary data_Revised.pdf]

membranes

Supplementary Materials

Tauroursodeoxycholic Acid (TUDCA) – Lipid Interactions and Antioxidant Properties of TUDCA Studied in Model of Photoreceptor Membranes

Michał Sabat [1], Anna M. Wiśniewska-Becker [1], Michał Markiewicz [2], Katarzyna Marzec [3], Jakub Dybaś [3], Justyna Furso [1], Paweł Pabisz [1], Mariusz Duda[1] and Anna M. Pawlak [1,*]

[1] Department of Biophysics, Faculty of Biochemistry, Biophysics and Biotechnology, Jagiellonian University, Krakow, Poland; michal.sabat@student.uj.edu.pl; anna.m.wisniewska@uj.edu.pl; justyna.furso@uj.edu.pl; pawel.pabisz@doctoral.uj.edu.pl; mariusz.z.duda@gmail.com

[2] Department of Computational Biophysics and Bioinformatics, Faculty of Biochemistry, Biophysics and Biotechnology, Jagiellonian University, Krakow, Poland; m.markiewicz@uj.edu.pl

[3] Jagiellonian Centre for Experimental Therapeutics, Jagiellonian University, Kraków, Poland; katarzyna.marzec@uj.edu.pl, jakub.dybas@uj.edu.pl

* Correspondence: anna.pawlak@uj.edu.pl

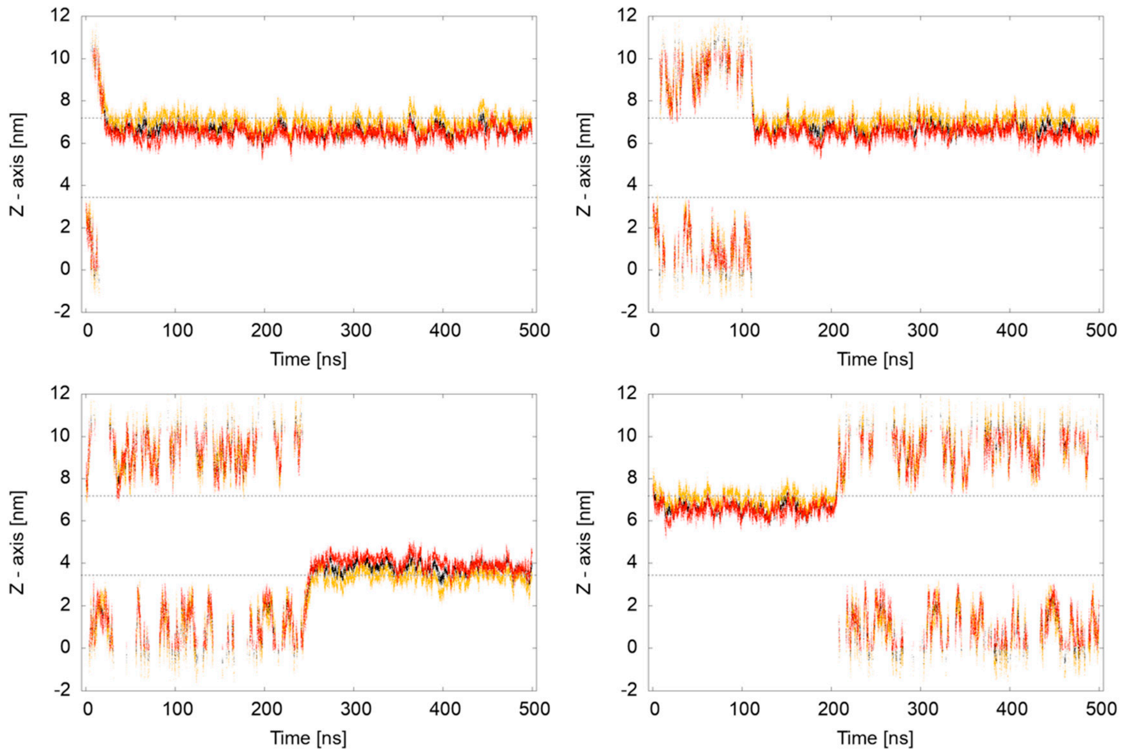

Figure S1. Z–coordinate plots for the center of mass (black line), sulfur (yellow) and O3 oxygen (red) atoms of four horizontally located molecules of TUDCA, which remained in a water phase for the entire simulation time. Dashed gray lines indicate the mean (from the entire simulation time) position of the P (phosphorus) atom of lipids.

membranes

MDPI

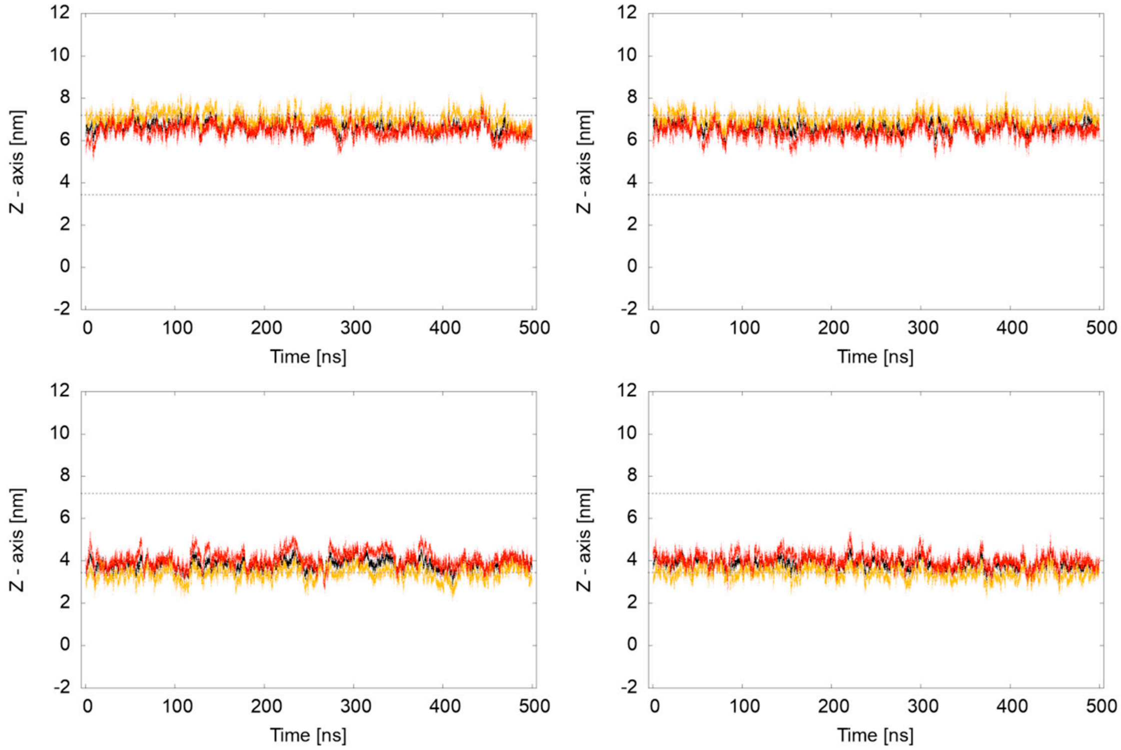

Figure S2. Z–coordinate plots for the center of mass (black line), sulfur (yellow) and O3 oxygen (red) atoms of four horizontally arranged molecules of TUDCA, which incorporated into the POPC membrane at different times of simulations. Dashed gray lines indicate the mean (from the entire simulation time) position of the P (phosphorus) atom of lipids.

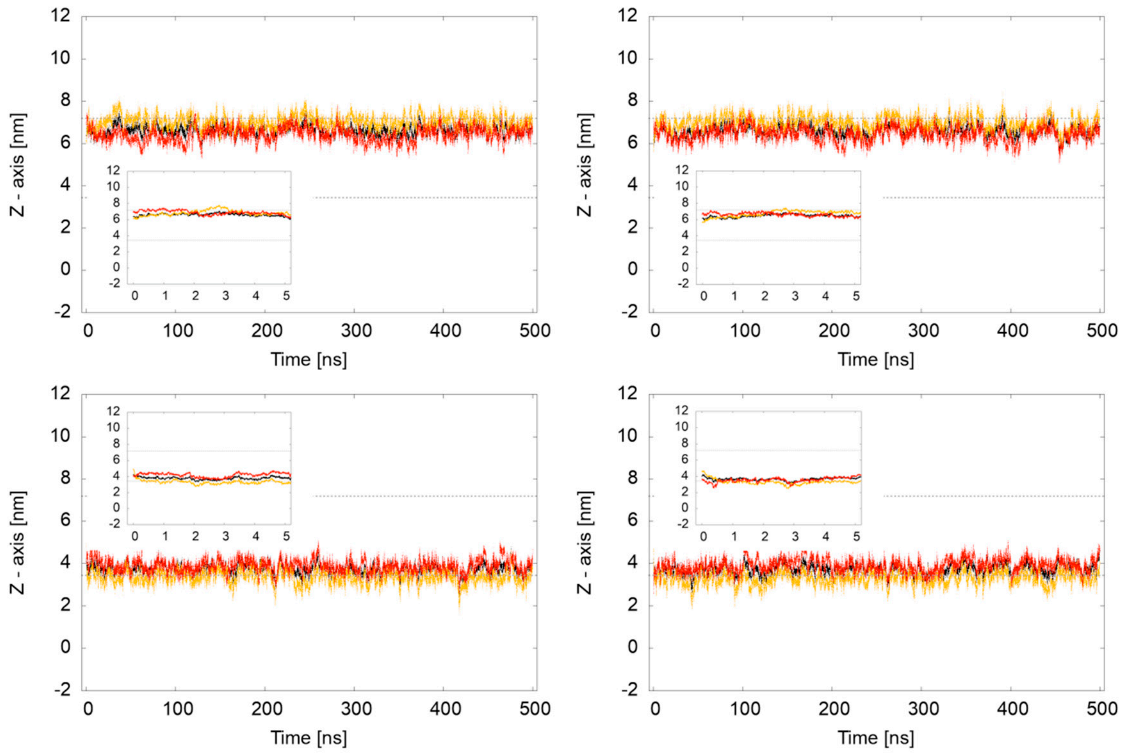

Figure S3. Z–coordinate plots for the center of mass (black line), sulfur (yellow) and O3 oxygen (red) atoms of four vertially located TUDCA molecules with sulphate group directed into the water phase. These molecules localized in the interphase region of POPC membrane. Dashed gray lines indicate the mean (from the entire simulation time) position of the P (phosphorus) atom of lipids

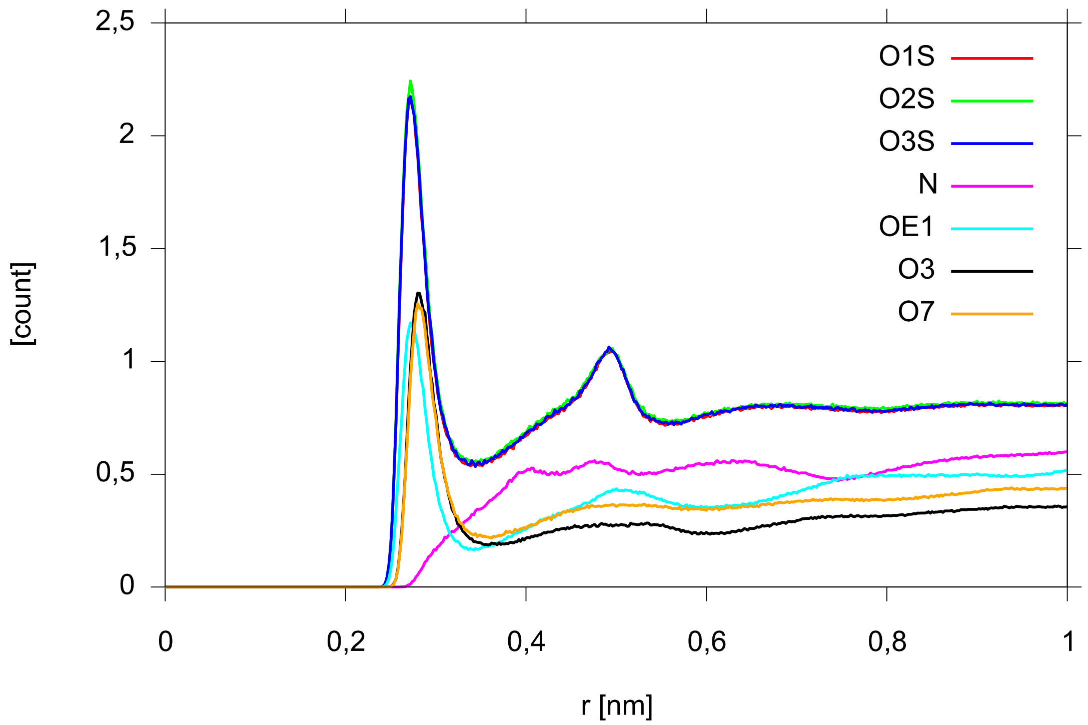

Figure S4 Radial distribution functions (RDFs) of water oxygen atoms relative to TUDCA oxygen atoms: O1S, O2S, O3S – oxygen atoms in sulphate group, OE1 – oxygen in carbonyl group and O3

and O7 – oxygen in the 3rd and 7th hydroxyl groups. Stronger ordering of water by the oxygens present in sulphate group than these of hydroxyl groups is evident.

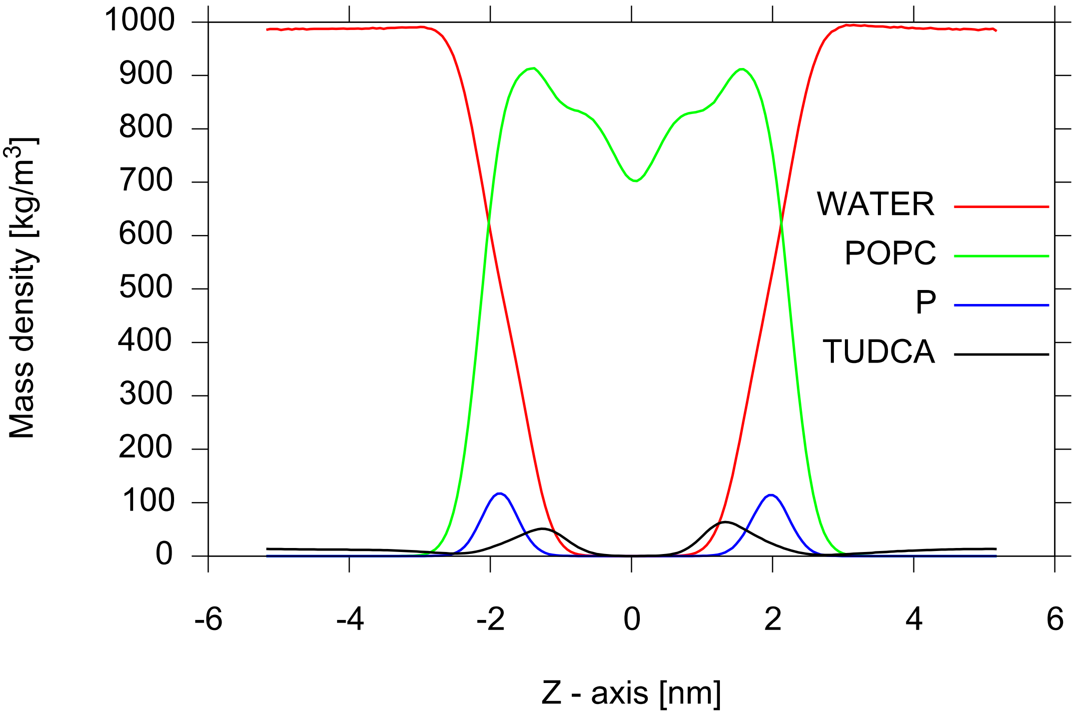

Figure S5 Mass density profiles along the bilayer normal (z axis, bilayer center at 0) of the atoms of water, lipids (POPC), phosphorous (P) and TUDCA (all 16 molecules).
